# Supplementary figures and images for: Self-organization of kinetochore-fibers in human mitotic spindles
Source: eLife. 2022 Jul 25;11:e75458. doi: 10.7554/eLife.75458 (PMC9398449; doi:10.7554/eLife.75458)

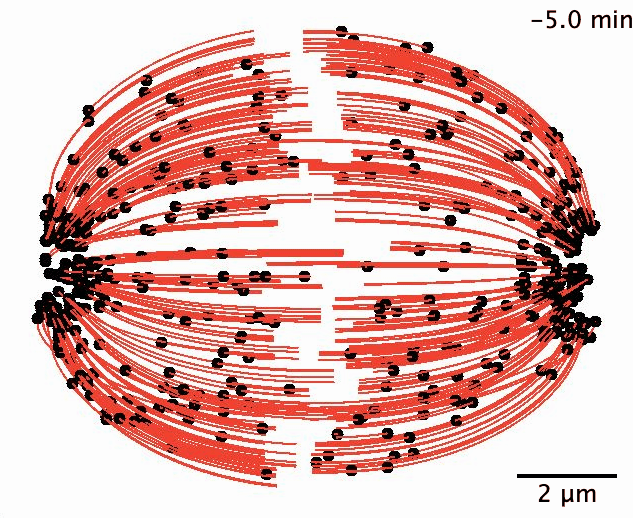

Supplement: Supplementary file 1 [file elife-75458-video1.gif]
